# Letter of Dr. T. C. Osborn

**Published:** 1885-08

**Authors:** T. C. Osborn

**Affiliations:** Cleburne, Tex.


### THE WAY THE DOCTORS DO IN ALABAMA.

CLEBURNE, TEX., July 20, 1885.

*Dear Doctor Daniel:*—The following was suggested by a merry rhyming letter sent me by Dr. Brigg, the Secretary of the Alabama State Medical Association in 1877, inclosing my credentials as a delegate to the Chicago meeting of the American Medical Association; and, as he was so pleased with its contents, I took care of the original draft for my children to see, after I am dead. The reason that prompts me to copy it for you is based upon the hope that it will not detract from the dignity of your new enterprise, and especially as it exhibits to the profession of this State the familiar courtesies of the profession of Alabama. I also feel sure there are a great number amongst your readers who will enjoy a farcical change from the grave composition embodied in the pages of the MEDICAL JOURNAL.

Your Friend, T. C. OSBORN.

For Daniel's Medical Journal.

GREENSBORO, ALA., June 13, 1876.

*My Dear Doctor Briggs:*—I write in much hurry,
Amidst the excitement and consequent flurry
Of getting off on the six o'clock train,
*En route* for the Medical Convention again.
But before leaving, I have concluded to say,
That you might look for, as sure as the day,

A note on arriving, all about the affair,
And all other oddities seen around there ;
By telegraph may be, or may be,by letter,
As my pocket may ever suggest as the better,
And cheaper; for, at best, all we can dare know,
In this, as the race—"the cash makes the mare go."

P. S.—June 6.

*Dear Doctor :*—The lines overhead were written on Sunday,
At night, preluding my departure on Monday ;
But while discussing the form of a beautiful conclusion
To the note, I found it, alas ! a most painful delusion.
Whilst thinking and yawning, the door knob sounded ;
Violent the peals were, and right up I bounded,
And in as great haste as I could to the door,
Ran blindly, and stumbling, fell flat on the floor,
Hitting my nose a great thump on its ridge,
Making millions of stars, each large as a midge.
And when I arose, after "cursing the luck,"
On opening the door wide, I found a big buck
Negro man, who most impatiently stated
That his wife was in labor, and possibly fated
To die in the agony, long before day,
Unless I would make haste and "took it away."
The night was quite dark, and so was the nigger ;
My nose was much swollen, and still growing bigger ;
And the fret from my fall, was cruelly crazing,
Making things that were small, seem hugely amazing.
But as I well knew there was pay in the job,
I hastily saddled my trusty old "Cob,"
And, mounting, paced alongside the man steadily,
Until we arrived there, finding the way readily.
By immediate assistance, in the course of an hour
The accouchement was over, and the man in my power.
Beyond doubt there is, on the earth to be found,
A balm and a solace sweet for each separate wound ;
My fee was ten dollars, which I readily cashed,
Which made me forget that my nose had been mashed.
Home again ; but my light breakfast not over,
When another call came ; so "feeling in clover,"

I strode off erect, forgetting the disaster,
My face wreathed in smiles, my nose in a plaster!
In a very short time, by specious analysis,
I diagnosed readily a case of pseudo paralysis.
The treatment was simple, my duty just clerical,
The work was on credit, and the woman hysterical!
After dinner, feeling dull, the temperature hot,
I doffed my old wrapper, and on the lounge got,
Resolved on the chance of an afternoon nap,
To make up for lost time, if no other mishap
Intervened to conflict with the sweetest of winks;
Then humming the air of the late "Captain Jinks,"
I lay at full length, with my eyelids fast falling—
Ah! there is a clatter, some one is calling—
"Doctor! oh, Doctor!" in tones quite distressing,
Which forced me to growl, and on with my dressing.
On looking around to learn what was the matter—
Thinking on credit and cash, but mostly the latter—
A sable messenger stated, "da sent me to beg
"You would come quick and splinter de leg
"Mr. Singly got broke, by a mighty bad kick
"Ob a mule, and he is now berry sick."
"Mr. Singly? oh! ah! yes," I am not at all miffy;
And feeling quite surgical, was off in a "jiffy."
Seven miles on the stretch, 'twas done in an hour;
Then handling the fracture the best in my power,
I adjusted the bones—tibia and fibula broken—
With bandages *en plaster*. And "by the same token,"
So delighted the patient by true deftness and skill,
That he pleasantly smiled when I made out my bill,
And "forked over" the sum in creditable cash—
Thirty-five dollars—all made at one dash!
Other cases came after, but of these I wont speak,
For to do so would encumber my time a whole week.
You know *now* the cause of the ugly embargo,
That prevented my pleasure of inhaling Chicago.
This mischance of going to the convention begets
Many bad feelings, perhaps painful regrets;
But honestly and candidly, I must frankly confess

That to do the thing over, I would do nothing less
Than I did. After all, perhaps, *you* in the end
Will lay no reproaches on me, my dear friend.
The state of my heart, though, is sad and forlorn,
But cordial as ever,

Yours Truly,

OSBORN.

P. S.

Convey in your next my regards—say everything nice,
—To that honorable gentleman, President Bryce;
And do not forget this, no, not for your life,
My profoundest esteem to his excellent wife.

